# Evaluating the Use of a Commercial Bacterial Biomass as a Bioactive Component of Pacific White Shrimp *Litopenaeus vannamei* Feeds

**DOI:** 10.1155/anu/8869967

**Published:** 2026-06-12

**Authors:** Magida Tabbara, Adela Araujo, Sidra Nazeer, Silvio Peixoto, Richard Smullen, Matthew Briggs, D. Allen Davis

**Affiliations:** ^1^ School of Fisheries, Aquaculture and Aquatic Sciences, Auburn University, Auburn, 36849, Alabama, USA, auburn.edu; ^2^ Fisheries Research and Training Institute, Manawan, Lahore, Pakistan; ^3^ Department of Fisheries and Aquaculture, Federal Rural University of Pernambuco, Recife, 52171–900, Pernambuco, Brazil, ufrpe.br; ^4^ Ridley Agriproducts Pty LTD, Level 9 South Tower Rialto 525 Collins Street, Melbourne, 3000, Victoria, Australia

**Keywords:** bacterial biomass, bioactive component, feed intake, functional feed additive, *Litopenaeus vannamei*, passive acoustic monitoring

## Abstract

Survival, growth rate, and welfare have always been the primary concerns of shrimp aquaculturists. Accordingly, a number of bioactive feed components have been supplemented in shrimp feed to improve health condition, feed intake, and growth. The present study evaluated the effects of supplementing a high ash, low protein marine bacterial biomass on feeding behavior, growth, proximate composition, hemolymph biochemistry, and digestive enzyme gene expression of juvenile *Litopenaeus vannamei*. Six isonitrogenous (36% crude protein) and isolipidic (6% crude lipid) diets were prepared to include graded levels (0%, 2.5%, 5%, 7.5%, 10%, and 20% of the diet) of a commercial bacterial biomass. Feed intake and behavior were assessed using passive acoustic monitoring prior to growth assessment. Following acoustic monitoring, juvenile *L. vannamei* (0.58 ± 0.02 g) were size‐sorted and stocked in an indoor recirculating aquaculture system. The experiment consisted of eight treatments, six of which were the diets prepared. The remaining two treatments were to offer shrimp the basal diet or the diet containing 10% bacterial biomass at 115% of the standard feeding ration. Each treatment consisted of five replicate aquaria, and the experiment was performed for 42 days. Results suggest that the inclusion of the bacterial biomass significantly affected shrimp feed consumption, growth, feed conversion ratio (FCR), alkaline phosphatase level, and protein and phosphorus retention (*p* < 0.05). Results were mainly influenced by bacterial biomass inclusion level. Shrimp offered the 10% bacterial biomass diet exhibited the most weight gain among shrimp offered the standard ration. However, offering shrimp the same diet but at 115% of the standard ration further improved weight gain. The present study suggests that supplementing shrimp feed with 10% of the commercial bacterial biomass we studied would lead to better growth, feed consumption, and feed conversion without adverse effects on hemolymph, overall health or digestion.

## 1. Introduction

There is considerable interest in developing diets that promote shrimp growth, survival, and good digestion. Modern aquaculture diets are formulated to meet the animals’ nutritional requirements in the most cost‐efficient manner [1]. Therefore, nutritionists adjust feed formulations primarily based on ingredient prices as the choice of ingredients dictates feed cost [2]. In aquaculture, there has been a shift towards using low‐fishmeal (FM) diets comprising higher inclusions of plant proteins. Soybean meal (SBM) became a popular plant protein source in aquaculture feed for a variety of reasons [3], mainly because of its balanced amino acid profile [4], moderate price, and relative availability at a steady supply [5]. However, such modifications in diets can impair animal growth and feed utilization [6, 7]. Decreased animal growth can result from the presence of antinutritional factors [8] and decreased palatability of the ingredient, as well as having inadequate levels of essential amino acids [9]. To compensate for the decrease in growth, certain feed additives can be incorporated into the feed formulation.

Feed additives are ingredients that can enhance feed quality and/or animal performance by modifying the physical or chemical characteristics of the feed [10]. Such additives can be synthetic or natural products [11] and come in various forms, including but not limited to prebiotics, probiotics, feed binders and effectors, organic acids, complex carbohydrates, and bioactive compounds [1, 12–14]. Bioactive compounds are of particular interest as they can improve aquaculture production by positively affecting animal growth and welfare [15]. Previous research suggested that using a certain bacterial biomass in low‐FM diets can help compensate for decreased shrimp growth [16, 17]. Such bacterial biomass can be considered a functional feed additive as it can help with shrimp growth [18, 19]. But in order to obtain the most cost‐effective feed formulation, it is important to evaluate the benefits of using such feed additives on shrimp performance from growth and physiology perspectives.

NovaqPro is a novel natural functional feed additive comprised of inactive bacteria and bioactive molecules [20]. The bacterial biomass is not a primary protein source but has a high ash content and is comprised of a myriad of inactivated and nongenetically modified beneficial bacteria and bioactive molecules. The bioactive molecules are mainly complex carbohydrates (including but not limited to lipopolysaccharides, polyhydroxybutyrate, uronic acid, etc.) trapped in an insoluble matrix [20]. Once in the foregut, intestinal enzymes help liberate the trapped molecules, which then stimulate the metabolism. The bacterial biomass seems to stimulate a variety of physiological aspects in organisms that consume it. According to Truong et al. [21], incorporating the bacterial biomass at 10% of the diet increases the feed intake of black tiger shrimp (*Penaeus monodon*). Similarly, Simon et al. [19] observed an increase in weight gain and a decrease in feed conversion ratio (FCR) when black tiger shrimp were offered feed with 10% of the same bacterial biomass. Mendoza‐Porras et al. [18] observed an upregulation of digestive enzymes in the hepatopancreas, explaining the observed improved use of dietary ingredients by shrimp. Based on the benefits obtained in black tiger shrimp, the present work evaluated the effects of using the high ash, low protein bacterial biomass on Pacific white shrimp juveniles’ feeding behavior, growth, and hemolymph biochemistry, as well as the expression of digestive enzymes and whole‐body composition.

## 2. Materials and Methods

### 2.1. Feed Preparation

A basal diet was formulated to contain 6% fishmeal, 46% SBM, and 8% corn protein concentrate as protein sources. Then, the basal diet was modified to contain 2.5%, 5%, 7.5%, 10%, and 20% of the bacterial biomass (Ridley Agriproducts Pty Ltd., Melbourne, Australia), allowing for a total of six experimental diets (Table 1). The proximate and amino acid compositions of the bacterial biomass are presented in Table 2. Experimental feeds were prepared in the aquatic animal nutrition laboratory of Auburn University, Auburn, AL, USA, using standard laboratory practices. Preground dry ingredients and oil were mixed thoroughly in a food mixer (Globe Food Equipment Co., Moraine, OH, USA). Afterwards, hot water (~40% w/w) was added to the mixture to form a mash with a more appropriate consistency. Each diet was then pressure‐pelleted using a meat grinder and a 3 mm die. The shrimp feed pellets obtained were dried to a moisture content of <10% in a forced‐air oven and stored at 4°C until use. The proximate and mineral composition of the experimental diets is presented in Table 3.

**Table 1:** Formulation of the experimental diets (as is).

Ingredients (%)	Basal	2.5% bacterial biomass	5% bacterial biomass	7.5% bacterial biomass	10% bacterial biomass	20% bacterial biomass
Fishmeal^a^	6.00	6.00	6.00	6.00	6.00	6.00
Soybean meal^b^	46.0	45.90	45.70	45.60	45.50	45.00
CPC^c^	8.00	8.00	8.00	8.00	8.00	8.00
Fish oil^a^	3.68	3.68	3.68	3.68	3.68	3.68
Soy oil	0.00	0.03	0.07	0.10	0.13	0.26
Lecithin^d^	1.00	1.00	1.00	1.00	1.00	1.00
Cholesterol^e^	0.15	0.15	0.15	0.15	0.15	0.15
Corn starch^f^	0.57	0.64	0.80	0.87	0.94	1.31
Whole wheat^g^	30.0	27.50	25.00	22.50	20.00	10.00
Mineral premix^h^	0.50	0.50	0.50	0.50	0.50	0.50
Vitamin premix^i^	1.80	1.80	1.80	1.80	1.80	1.80
Choline chloride^j^	0.20	0.20	0.20	0.20	0.20	0.20
Stay‐C^k^	0.10	0.10	0.10	0.10	0.10	0.10
CaP‐dibasic^l^	2.00	2.00	2.00	2.00	2.00	2.00
Bacterial biomass^m^	0.00	2.50	5.00	7.50	10.00	20.00

^a^Omega Protein Inc., Reedville, VA, USA.

^b^Dehulled solvent‐extracted soybean meal, Bunge Limited, Decatur, AL, USA.

^c^Corn protein concentrate, Empyreal 75, Cargill Corn Milling, Cargill, Inc, Blair, NE, USA.

^d^The Solae Company, St. Louis, MO, USA.

^e^Alfa Aesar, Haverhill, MA, USA.

^f^MP Biomedicals, Solon, OH, USA.

^g^Bob’s Red Mill, Milwaukie, OR, USA.

^h^Trace mineral premix (g/100 g premix): cobalt chloride, 0.004; cupric sulfate pentahydrate, 0.550; ferrous sulfate, 2.000; magnesium sulfate anhydrous, 13.862; manganese sulfate monohydrate, 0.650; potassium iodide, 0.067; sodium selenite, 0.010; zinc sulfate heptahydrate, 13.193; and alpha‐cellulose, 69.664.

^i^Vitamin premix (g/kg premix): thiamin HCL, 4.95; riboflavin, 3.83; pyridoxine HCL, 4.00; Ca‐pantothenate, 10.00; nicotinic acid, 10.00; biotin, 0.50; folic acid, 4.00; cyanocobalamin, 0.05; inositol, 25.00; vitamin A acetate (500,000 IU/g), 0.32; vitamin D3 (1,000,000 IU/g), 80.00; menadione, 0.50; and alpha‐cellulose, 856.81.

^j^VWR International, Radnor, PA, USA.

^k^Stay *C*, (L‐ascorbyl‐2‐polyphosphate 35% Active C), Roche Vitamins Inc., Parsippany, NJ, USA.

^l^Beantown Chemical, Hudson, NH, USA.

^m^NovaqPro, Ridley Agriproducts Pty Ltd., Level 9, South Tower Rialto, 525 Collins Street, Melbourne VIC 3000, Australia.

**Table 2:** Proximate and amino acid composition of the bacterial biomass used in the present work (as‐is basis).

Proximate composition (%)	
Moisture	21.04
Crude protein	16.58
Crude fat	0.38
Ash	40.93
Amino acid composition (%)	
Alanine	0.92
Arginine	0.53
Aspartic acid	1.57
Cysteine	0.19
Glutamic acid	1.52
Glycine	0.90
Histidine	0.17
Hydroxylysine	0.02
Hydroxyproline	0.02
Isoleucine	0.55
Lanthionine	0.03
Leucine	0.81
Lysine	0.50
Methionine	0.16
Ornithine	0.06
Phenylalanine	0.57
Proline	0.50
Serine	0.63
Taurine	0.02
Threonine	0.73
Tryptophan	0.11
Tyrosine	0.39
Valine	0.79

**Table 3:** Proximate and mineral composition of the experimental diets (dry matter basis).

Components	Basal	2.5% bacterial biomass	5% bacterial biomass	7.5% bacterial biomass	10% bacterial biomass	20% bacterial biomass
Proximate composition (%)						
Moisture	6.03	6.93	6.18	7.03	9.02	6.78
Crude protein	40.6	40.5	40.4	40.6	41.1	40.8
Crude fat	6.81	6.77	7.44	7.06	6.88	6.86
Ash	7.32	8.23	9.05	10.6	11.4	15.2
Mineral composition						
Sulfur (%)	0.42	0.43	0.45	0.5	0.51	0.59
Phosphorus (%)	1.17	1.26	1.34	1.56	1.62	1.92
Potassium (%)	1.32	1.42	1.3	1.28	1.36	1.39
Magnesium (%)	0.22	0.26	0.34	0.43	0.48	0.73
Calcium (%)	1.19	1.17	1.43	1.71	1.78	2.22
Sodium (%)	0.10	0.28	0.38	0.61	0.72	1.3
Iron (ppm)	468	248	239	291	297	371
Manganese (ppm)	54.0	53.1	58.7	59.6	64.7	73.7
Copper (ppm)	19.6	15.7	16.2	18.9	21.8	19.4
Zinc (ppm)	119	119	122	126	115	144

*Note:* Proximate and mineral analysis was performed in Midwest Laboratories Inc., following AAFCO guidelines (Omaha, NE, USA).

### 2.2. Feed Consumption and Acoustic Activity

Considering that the recommended commercial dose of bacterial biomass inclusion is between 5% and 10%, evaluation of dry matter loss, feed consumption, and acoustic recordings were performed using the basal, 5%, and 10% bacterial biomass diets only. The dry matter loss of the feed submerged in the water was determined first. 1 g of feed (on an as‐is basis) was dispensed in the center of 12 replicate 80 L glass aquaria. After 30 min, the feed was siphoned and collected onto preweighed dry cellulose filters of 20 μm pore size. The leftover collected feed and filters were dried overnight in an oven at 100°C and then weighed. Afterwards, 10 shrimp (average individual weight = 2.72 ± 0.50 g) were transported from our biofloc nursery tank and stocked into 12 glass aquaria. Diets were randomly assigned to the aquaria, allowing for four replicate aquaria per diet. Shrimp were acclimated to the experimental diets for at least 48 h prior to determining feed consumption and evaluating acoustic activity. Feed consumption was determined by offering shrimp 1 g of feed (on an as‐is basis) and then recovering leftover unconsumed feed after 30 min, as described previously. Feed consumption (FC, in g) was calculated on a dry matter basis

FC=Fo–Fr×Fl,

where FC = feed consumption (g), *F*_o_ = feed offered (g), *F*_*r*_ = recovered feed (g), and *F*_l_ = leached feed (g) calculated as

Fl=Fr/Fo.

Evaluation of acoustic activity was performed in tandem with the determination of feed consumption, following the same protocol used in Peixoto et al. [22, 23], Soares et al. [24], and Tabbara et al. [25]. Acoustic activity in terms of shrimp mandible clicks was recorded using omnidirectional AS‐1 hydrophones (Aquarian Hydrophones, Anacortes, WA, USA). The linear frequency of the hydrophones ranged between 1 Hz and 100 kHz, and the receiving sensitivity was 208 dBV. One hydrophone was placed in the center of each aquarium, 15 cm from the bottom. Then, every two hydrophones were connected to a two‐channel multitrack recorder (H5 Audio Recorder, Zoom North America LLC, Hauppauge, NY, USA), whose sampling frequency was 96 kHz using preamplifiers with 26 dB gain (PA‐4, Aquarian Hydrophones, Anacortes, WA, USA). The recording started when the feed was dropped in the aquarium and continued for a total of 30 min. During the recording, aeration and water circulation were discontinued to reduce background noise. Collected acoustic data were analyzed using Raven Pro 1.6 (Raven Sound Analysis, Cornell Lab of Ornithology, Cornell University, NY, USA).

### 2.3. Growth Evaluation

Pacific white shrimp (*Litopenaeus vannamei*) postlarvae were purchased from a commercial hatchery (HomeGrown Shrimp, Indiantown, FL, USA) and raised in a biofloc nursery tank at the E. W. Shell Fisheries Center of Auburn University (Auburn, AL, USA) until used in the experiment. Fifteen Pacific white shrimp juveniles (average individual weight = 0.58 ± 0.02 g) were size sorted for uniformity, group‐weighed, and stocked in an indoor recirculating aquaculture system comprised of a battery of 132 L glass aquaria. All aquaria were connected to a common biological filter, a physical bead filter (Aquadyne Filtration Systems, Hartwell, GA, USA), and a common sump. Aside from the six diets prepared, two additional dietary treatments were added to the experiment based on preliminary data (data not published). The additional treatments consisted of providing feed (basal diet and diet with 10% bacterial biomass) in excess of the standard feeding ration by 15%. The treatments were randomly assigned to the aquaria, allowing for five replicates.

During the growth evaluation, shrimp were manually offered feed four times a day (8:00, 11:00, 13:00, and 17:00 o’clock) using a standardized feeding table that is based on the historical results from our laboratory. Feed inputs were calculated presuming shrimp will double in weight weekly up to 1 g and then gain 0.8 g per week, with a presumed FCR of 1.8%. Daily feed inputs were adjusted every week based on shrimp counts as well as observations of leftover feed. In general, shrimp were offered feed in slight excess to avoid underfeeding based on preliminary data. After 42 days, the experiment was concluded, and shrimp in each tank were counted and group‐weighed to determine the survival, weight gain, and FCR.

### 2.4. Hemolymph Biochemical Analysis

Four shrimp were randomly selected from each aquarium for hemolymph collection. 1‐cc syringes equipped with 25‐gauge needles were used to withdraw hemolymph from the pericardial cavity of shrimp, following the protocol of Roy et al. [26]. To prevent clotting, the syringes were prefilled with an anticoagulant prepared according to Liu et al. [27]. The quantity of anticoagulant was predetermined to roughly equate to the quantity of hemolymph expected (1:1 ratio, on a weight basis) and to allow for the calculation of a dilution factor. The collected hemolymph samples were centrifuged at 1000 ×*g* for 15 min (Marathon 16 km, Fisher Scientific, Waltham, MA, USA). Then, the hemolymph samples were used to determine alkaline phosphatase, alanine aminotransferase, γ glutamyl transferase, urea nitrogen, and cholesterol using a VetScan VS2 Chemistry Analyzer (Abaxis, Inc., Union City, CA, USA).

### 2.5. Digestive Enzyme Gene Expression Analysis

Afterwards, three shrimp were randomly selected from each aquarium and dissected. The hepatopancreas of those shrimp was extracted and stored in a DNA/RNA Shield (Zymo Research, Orange, CA, USA). Then, RNA was extracted from the hepatopancreas using the Quick‐RNA MiniPrep Plus kit (Zymo Research, Orange, CA, USA) following the recommended instructions. Afterwards, RNA quality and concentration were assessed using a microvolume UV‐Vis spectrophotometer (NanoDrop One/One^C^, Thermo Scientific, Waltham, MA, USA). Subsequently, extracted RNA was diluted to a final concentration of 50 ng/mL before being synthesized into cDNA using the High‐Capacity cDNA Reverse Transcription Kit (Applied Biosystems, Waltham, MA, USA). The total volume of each reaction well was 20 μL, which comprised 2 μL of 10 × RT buffer, 0.8 μL of 25 × dNTP mix, 2 μL of 10 × random primers, 1 μL of multiscribe reverse transcriptase, 4.2 μL of nuclease‐free water, and 10 μL of diluted RNA. cDNA was synthesized using a thermal cycler (T100 Thermal Cycler, Bio‐Rad Laboratories, Inc., Hercules, CA, USA) with the following parameters set for the thermal program: denaturation for 10 min at 25°C, annealing for 120 min at 37°C, and extension for 5 min at 85°C. The obtained cDNA with a concentration of 9 ng/μL was further diluted to a final concentration of 1.25 ng/μL before use to evaluate the expression of *trypsin* and *chymotrypsin*. *Ubiquitine* was used as the reference gene (Table 4).

**Table 4:** Pacific white shrimp primers used in the present work to evaluate digestive enzyme gene expression.

Gene	Forward primer sequence	Reverse primer sequence	References
*Trypsin*	TCCAAGATCATCCAACACGA	GACCCTGAGCGGGAATATC	Xie et al. [28]
*Chymotrypsin*	GGCTCTCTTCATCGACG	CGTGAGTGAAGAAGTCGG	Xie et al. [28]
*Ubiquitine*	GGGAAGACCATCACCCTTG	TCAGACAGAGTGCGACCATC	Ulaje et al. [29]

Quantitative real‐time PCR was performed using a QuantStudio 5 Real‐Time PCR system (Applied Biosystems, Waltham, MA, USA). Each reaction well contained 5 μL of PowerUp SYBR Green Master Mix (Applied Biosystems, Waltham, MA, USA), 0.5 μL of forward and 0.5 μL of reverse primers, and 4 μL of the previously diluted cDNA. Each sample was analyzed in duplicates, and the negative control used consisted of nuclease‐free water. The cycle parameters were set to 2 min at 50°C, then 2 min at 95°C, followed by 15 s at 95°C, 15 s at 58°C, and 30 s at 72°C for a total of 40 cycles. The abundance of genes of interest was calculated in the end, following the 2^-ΔΔCt^ method [30].

### 2.6. Whole Body Proximate Analysis

At the end of the growth evaluation, four shrimp per tank were euthanized and stored at −20°C for whole‐body proximate analysis. Subsequently, shrimp were dried in an oven at 100°C until a stable weight was obtained. Proximate analysis was subsequently performed on a dry matter basis at Midwest Laboratories Inc., following AAFCO guidelines (Omaha, NE, USA).

### 2.7. Water Quality

Water quality parameters in terms of dissolved oxygen, salinity, temperature, pH, total ammonia nitrogen (TAN), and nitrite nitrogen were maintained within good ranges for Pacific white shrimp. Dissolved oxygen (mg/L), temperature (°C), and salinity (g/L) were measured twice a day using a handheld meter (YSI Pro2030, YSI Inc., Yellow Springs, OH, USA). pH was measured twice a week using a handheld pH meter (PH110: ExStik Refillable pH Meter, Extech Instruments, Nashia, NH, USA). TAN (mg/L) and nitrite nitrogen (mg/L) were measured twice a week using an economical photometer (YSI 9500, YSI Inc., Yellow Springs, OH, USA) (Table 5).

**Table 5:** Water quality parameters of the experimental system used during the growth evaluation for 42 days (Mean ± SD).

Parameter	
Dissolved oxygen (mg/L)	7.22 ± 0.30
Salinity (g/L)	9.55 ± 0.14
Temperature (°C)	28.67 ± 0.46
pH	7.68 ± 0.57
TAN (mg/L)	0.32 ± 0.27
Nitrite‐N (mg/L)	0.07 ± 0.04

### 2.8. Data Analysis and Statistics

Statistical analyses were performed using the SAS (V9.4, SAS Institute, Cary, NC, USA). All data were reported as mean values of the replicates and tested for normality, homoscedasticity, and homogeneity of variance using Shapiro–Wilk [31], White [32], and Bartlett [33] tests, respectively. Feed consumption data were analyzed using a two‐sample *t*‐test, whereas for acoustic activity data, regression analysis was used to analyze the relationship between the clicking frequency and time. Gene expression data were log‐transformed to meet normality assumptions [34]. Growth, whole‐body proximate composition, hemolymph biochemistry, and gene expression data were analyzed using one‐way analysis of variance (ANOVA). Differences were considered significant at *p* < 0.05 and were analyzed using Student‐Newman‐Keuls’ post hoc test. A two‐way ANOVA was also used to analyze growth, whole‐body proximate composition, and hemolymph biochemistry of shrimp offered standard and excess rations of the basal and the 10% bacterial biomass diet. Figures were produced using GraphPad Prism (v10.1.0; GraphPad Software, Inc., Boston, MA, USA).

## 3. Results

### 3.1. Feed Consumption and Acoustic Activity

**Figure 1:**
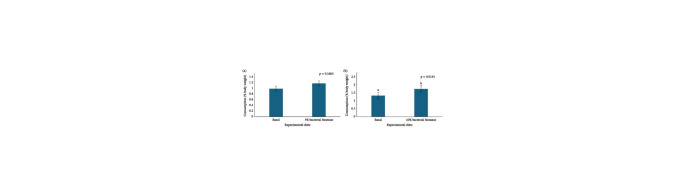
Quantity of feed consumed as a percentage of body weight of Pacific white shrimp juveniles offered the basal diet and diet containing 5% bacterial biomass or 10% bacterial biomass. Bars represent the mean feed consumed as a percentage of body weight after 30 min, and error bars represent the standard error of the mean. (a) Series 1 and (b) Series 2.

Results of the present work indicate that incorporating the bacterial biomass at 10% of the shrimp diet significantly increased feed consumption (*p* = 0.0141; Figure 1). Shrimp offered the diet containing 10% bacterial biomass consumed feed equivalent to 1.75% of body weight, whereas shrimp from the same trial series but offered the basal diet consumed the equivalent of 1.33% of body weight. A similar trend was observed with shrimp offered the diet containing 5% bacterial biomass, which consumed feed equivalent to 1.18% of body weight, compared to the ones offered the basal diet, which consumed feed equivalent to only 0.99% of body weight.

**Figure 2:**
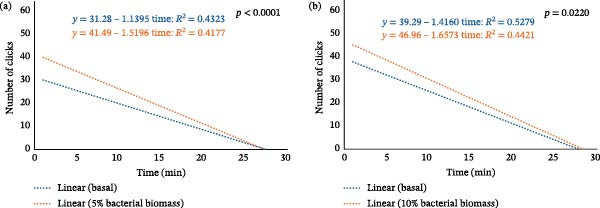
Linear regression between the number of clicks emitted by Pacific white shrimp juveniles offered basal diet and diet containing 5% bacterial biomass (a) or basal diet and 10% bacterial biomass (b) over time (in minutes).

Regression analysis of the acoustic data over time indicates a significant effect of the supplement on feed intake (*p* < 0.05; Figure 2). Shrimp offered the diet containing 5% bacterial biomass emitted significantly more clicks than shrimp offered the basal diet (*p* < 0.0001) over the 30 min period of recording. Similarly, shrimp offered the 10% bacterial biomass diet emitted significantly more clicks than shrimp offered the basal diet (*p* = 0.0220) throughout the duration of the recording. Acoustic data support the feed consumption results.

### 3.2. Growth Evaluation

Results of the present work showed significant differences in terms of the final biomass of shrimp offered the various experimental diets at the standard feeding ration (*p* = 0.0012; Table 6). The shrimp final biomass seemed to significantly improve after including 5%, 7.5%, or 10% bacterial biomass, with the biggest biomass being recorded for shrimp offered the 5% bacterial biomass diet. However, the shrimp’s final weight, weight gain, and survival were not significantly influenced by the inclusion of the bacterial biomass when the diets were offered at the standard feeding ration. The final average individual weight ranged between 7.26 g and 8.57 g (shrimp offered the basal and 10% bacterial biomass diets, respectively), whilst weight gain varied between 1206.20% and 1362.37% (basal and 10% bacterial biomass diets, respectively). The overall survival of shrimp was 83.83%, with no significant differences among treatments. FCR significantly differed among shrimp offered the various diets, with shrimp offered the basal diet having a significantly bigger FCR (1.63) than shrimp offered the 10% bacterial biomass diet (1.34).

**Table 6:** Growth performance of Pacific white shrimp juveniles (average initial weight = 0.58 ± 0.02 g) offered various diets containing graded levels of bacterial biomass in addition to two diets with overage ration for 42 days (*n* = 5).

Treatment	Final biomass (g)	Final weight (g)	Weight gain (%)	Survival (%)	FCR
Basal	86.03^b^	7.26^b^	1206.20^ab^	80.00	1.63^b^
2.5% bacterial biomass	100.80^ab^	8.03^ab^	1290.14^ab^	84.00	1.45^bc^
5% bacterial biomass	111.59^a^	8.21^ab^	1292.55^ab^	90.67	1.42^bc^
7.5% bacterial biomass	109.60^ab^	7.88^ab^	1286.67^ab^	93.33	1.49^bc^
10% bacterial biomass	107.45^ab^	8.57^ab^	1362.37^ab^	84.00	1.34^c^
20% bacterial biomass	86.64^b^	7.75^b^	1235.98^ab^	76.00	1.54^bc^
Basal 115% ration	84.70^b^	7.21^b^	1180.52^b^	78.67	1.92^a^
10% bacterial biomass 115% ration	114.32^a^	9.11^a^	1451.87^a^	84.00	1.44^bc^
PSE	5.8322	0.3134	57.6371	5.8403	0.0649
*P* value	0.0012	0.0023	0.0516	0.4468	<0.0001
Two‐way ANOVA (Type III SS)					
% bacterial biomass	0.0014	<0.0001	0.0006	0.4921	<0.0001
Feed ration	0.6814	0.3150	0.5371	0.9213	0.0018
% bacterial biomass × feed ration	0.5442	0.2335	0.2717	0.9213	0.0841

*Note:* Values within the same column bearing different superscripts differ significantly from each other (*p* < 0.05).

When offered diets in excess of the standard feeding ration, significant differences were observed in terms of final biomass, final weight, weight gain, and FCR (*p* < 0.05; Table 5). Shrimp growth was significantly better when offered the 10% bacterial biomass diet, regardless of the ration. The shrimp’s final weight was significantly bigger when offered the 10% bacterial biomass at the 115% ration compared with the basal diet at the same ration (9.11 g and 7.21 g, respectively). Weight gain followed a similar trend to final weight, where shrimp offered the 10% bacterial biomass at 115% ration gained weight by 1451.87% compared with shrimp offered the basal diet at the same ration, where weight gain was only 1180.52%. FCR was significantly influenced by the bacterial biomass inclusion level (*p* < 0.0001) as well as the ration offered (*p* = 0.0018). Shrimp offered the basal diet had significantly bigger FCRs than shrimp offered the 10% bacterial biomass diet. Interestingly, FCRs of shrimp offered the 10% bacterial biomass diet at either ration did not differ significantly. Survival was not significantly different between both diets (*p* > 0.05).

### 3.3. Hemolymph Biochemical Analysis

Results of the analysis of hemolymph of shrimp offered diets with increasing levels of bacterial biomass indicated significant differences in terms of alkaline phosphatase (*p* = 0.0012; Table 7). Shrimp offered a diet including 2.5% bacterial biomass had a significantly higher alkaline phosphatase level than shrimp‐offered diets containing 10% and 20% bacterial biomass (192.17 U/L for shrimp offered 2.5% bacterial biomass compared to 106.58 U/L and 65.32 U/L for shrimp offered 10% and 20% bacterial biomass, respectively). However, no significant differences were observed in terms of levels of alanine aminotransferase, gamma glutamyl transferase, urea nitrogen, or cholesterol of shrimp offered the various diets (*p* > 0.05).

**Table 7:** Hemolymph biochemical analysis of Pacific white shrimp juveniles (average initial weight = 0.58 ± 0.02 g) offered various diets containing graded levels of bacterial biomass in addition to two diets with overage ration for 42 days (*n* = 5).

Treatment	Alkaline phosphatase (U/L)	Alanine aminotransferase (U/L)	γ Glutamyl transferase (U/L)	Urea nitrogen (mg/dL)	Cholesterol (mg/dL)
Basal	138.78^abc^	99.20	2.48	4.69	93.10
2.5% bacterial biomass	192.17^a^	118.32	3.34	4.37	91.86
5% bacterial biomass	150.57^ab^	95.71	6.66	4.24	66.80
7.5% bacterial biomass	135.97^abc^	186.11	4.16	3.33	104.52
10% bacterial biomass	106.58^bc^	106.65	4.88	4.15	118.68
20% bacterial biomass	65.32^c^	122.73	4.81	4.24	86.71
Basal 115% ration	152.26^ab^	84.62	2.82	4.31	74.75
10% bacterial biomass 115% ration	93.00^bc^	97.84	3.02	4.00	69.87
PSE	18.4610	40.2668	1.1294	0.6063	13.4523
*P* value	0.0012	0.7361	0.1944	0.8851	0.1337
Two‐way ANOVA (Type III SS)					
% bacterial biomass	0.0074	0.5099	0.0683	0.5222	0.4624
Feed ration	0.9974	0.4569	0.2720	0.6878	0.0265
% bacterial biomass × feed ration	0.3780	0.8531	0.1175	0.8647	0.2838

*Note:* Values within the same column bearing different superscripts differ significantly from each other (*p* < 0.05).

### 3.4. Digestive Enzyme Gene Expression Analysis

**Figure 3:**
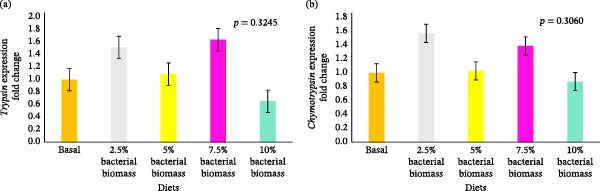
Trypsin (a) and chymotrypsin (b) enzymes gene expression of Pacific white shrimp juveniles (average initial weight = 0.58 ± 0.02 g) offered various diets containing graded levels of bacterial biomass for 42 days (*n* = 3). Bar graphs represent mean values of the replicates of relative expression (2^−ΔΔCt^), and the error bar represents the standard error. Statistical analysis was performed on log‐transformed data.

Analysis of *trypsin* and *chymotrypsin* activity in the hepatopancreas of shrimp offered the various diets did not result in significant differences (Figure 3). Expression of *trypsin* seemed to vary among shrimp offered the various diets, with the gene being most expressed in shrimp offered 2.5% and 7.5% bacterial biomass. Similar trends were observed in terms of *chymotrypsin* expression, with the gene being most expressed in the hepatopancreas of shrimp offered 2.5% and 7.5% bacterial biomass.

### 3.5. Whole Body Proximate Analysis

Inclusion of bacterial biomass in shrimp diets did not significantly influence whole‐body proximate composition (*p* > 0.05; Table 8). Whole body dry matter ranged between 25.72% and 27% (basal and 10% bacterial biomass diets, respectively), whereas whole‐body protein ranged between 77.20% and 78.54% (2.5% and 10% bacterial biomass diets, respectively). Additionally, shrimp whole‐body fat varied between 10.43 and 11.12 (10% bacterial biomass and basal diet, respectively), whereas whole‐body ash varied between 9.41 and 10.22 (basal and 20% bacterial biomass, respectively). The highest levels of dry matter and protein were noted in shrimp that were offered 10% bacterial biomass at a 115% ratio (28.77% and 79.04%, respectively).

**Table 8:** Whole body proximate composition, apparent net protein retention, and phosphorus retention of Pacific white shrimp juveniles (average initial weight = 0.58 ± 0.02 g) offered various diets containing graded levels of bacterial biomass in addition to two diets with an overage ration for 42 days (*n* = 5).

Treatment	Dry matter (%)	Crude protein (%)	Crude fat (%)	Ash (%)	ANPR (%)	Phosphorus retention (%)
Basal	25.72	77.26	11.12	9.41	32.44^ab^	13.45^ab^
2.5% bacterial biomass	25.86	77.20	10.60	9.88	35.60^ab^	14.56^ab^
5% bacterial biomass	26.54	78.10	10.90	9.44	38.08^ab^	15.38^ab^
7.5% bacterial biomass	26.57	77.50	11.02	10.17	35.85^ab^	13.96^ab^
10% bacterial biomass	27.00	78.54	10.43	10.17	40.24^a^	15.77^a^
20% bacterial biomass	26.45	77.44	10.92	10.22	35.01^ab^	12.22^ab^
Basal 115% ration	26.76	76.72	11.06	9.54	28.38^b^	12.03^b^
10% bacterial biomass 115% ration	28.77	79.04	10.49	9.94	40.57^a^	15.40^ab^
PSE	1.1250	1.0411	0.4506	0.2298	2.2904	0.8124
*P* value	0.6774	0.7905	0.9160	0.0625	0.0125	0.0119
Two‐way ANOVA (Type III SS)						
% bacterial biomass	0.2338	0.1213	0.2376	0.0229	0.0019	0.0075
Feed ration	0.3075	0.9857	0.9939	0.8412	0.4994	0.3484
% bacterial biomass × feed ration	0.7867	0.6428	0.9030	0.4543	0.4270	0.5819

*Note:* Values within the same column bearing different superscripts differ significantly from each other (*p* < 0.05).

Significant differences were noted in terms of apparent net protein retention (ANPR) of shrimp offered the various diets (*p* = 0.0125). Shrimp‐offered 10% bacterial biomass retained protein the most (ANPR = 40.24%) compared with shrimp offered the basal diet, which seemed to retain the least protein (ANPR = 32.44%). Shrimp offered the basal diet at 115% of the ration retained significantly less protein than shrimp offered any of the other diets (ANPR = 28.28%). The variation in protein retention is mainly attributed to the level of bacterial biomass inclusion (*p* = 0.0019) as opposed to the feed ration (*p* > 0.05).

Phosphorus retention data followed similar trends to ANPR data and were significantly influenced by the inclusion of bacterial biomass in the diets (*p* = 0.0119). Among shrimp offered the standard feeding ration, phosphorus retention varied between 12.22% and 15.77% (20% and 10% bacterial biomass diets, respectively). However, a significant decrease in phosphorus retention was observed among shrimp offered the basal diet at an 115% ration (12.03%) in comparison with shrimp offered the 10% bacterial biomass diet at the standard ration (15.77%). The variation in phosphorus retention was mainly associated with the level of bacterial biomass in the diets (*p* = 0.0075) as opposed to the feed ration (*p* > 0.05).

## 4. Discussion

### 4.1. Feed Consumption and Acoustic Activity

Acoustic activity data collected from the present work indicated that the inclusion of bacterial biomass in the diet positively affects feed consumption. Despite not being statistically significant, the total number of clicks emitted from shrimp offered the basal diet was smaller than the number of clicks emitted from shrimp offered diets containing 5% or 10% bacterial biomass. The feed consumption data corroborate the acoustic activity results as feed consumption seemed to increase with the increased level of bacterial biomass in the diet. Information obtained in terms of the total number of clicks emitted and supported by the feed consumption data suggest that the bacterial biomass can improve the chemical properties of the feed, making it more attractive and palatable. Glencross et al. [16] observed a similar increase in feed intake of black tiger shrimp when offered low‐FM diets containing bacterial biomass at 10%.

Acoustic activity data also indicated that shrimp offered any of the three diets tended to consume feed quickly once offered, and then click frequency tended to decrease 15 min later. Previous research also noted a similar observation, suggesting that offering feed to fasted shrimp results in voraciousness [23, 25, 35, 36]. However, the number of clicks emitted when the basal diet was offered was the smallest. The number of clicks seemed to increase with the increase in the level of bacterial biomass in the diet. Such observations, together with the total number of clicks and the feed consumption data, further indicate that the bacterial biomass can act as a feed effector, increasing feed palatability. Shrimp depend on chemoreceptors to locate their feed [37]. If offered palatable and chemically attractive feed, shrimp tend to locate feed pellets faster [38], which results in faster feed consumption [39]. One of the benefits of using such a feed effector would be to induce faster feed location and consumption, which minimizes nutrient leaching [40], which in turn participates in minimizing expenditures.

### 4.2. Growth Evaluation

The results of the present work suggest that incorporating the bacterial biomass in shrimp diets can significantly improve growth. An overall increase in weight gain is observed in shrimp offered diets containing the bacterial biomass, with the biggest gain in weight being observed in shrimp offered the diet with 10% bacterial biomass, regardless of the feed ration. The increased weight gain can be attributed to the presence of bacterial biomass in the feed. As observed from the acoustic activity data, shrimp seemed to locate and consume bacterial biomass‐containing feed faster than the basal diet. Such activity minimizes the time the feed stays in the water, which in turn minimizes nutrient leaching from the feed. Therefore, shrimp offered feed containing the bacterial biomass could benefit more from the dietary nutrients compared to shrimp offered the basal diet. Additionally, shrimp offered diets containing the bacterial biomass seemed to consume more food. The increase in feed consumption allows for better growth, especially when the feed is formulated to meet the animals’ dietary requirements [41]. The FCR values obtained reinforce the idea that shrimp seemed to have made better use of dietary nutrients when offered diets containing the bacterial biomass. Shrimp‐offered feed containing 10% bacterial biomass exhibited the biggest weight gain and the smallest FCR, which explains the significant improvements in nutrient assimilation. Our results are in agreement with Rombenso et al. [42], who also observed significant improvements in final weight and percent weight gain of Pacific white shrimp juveniles offered a low‐FM diet with 10% bacterial biomass compared to the control diet.

Previous research evaluating the use of bacterial biomass in shrimp feed assessed the effects of the bacterial biomass on shrimp growth when offered a restricted feed ration or feed in excess. According to Simon et al. [19], juvenile *Penaeus monodon* exhibited significantly better weight gain and FCR when offered feed containing 10% bacterial biomass at 120% of the standard ration. Our results are in agreement with Simon et al. [19], as a significant increase in weight gain was observed between shrimp offered the basal diet and those offered the 10% bacterial biomass diet, both at 115% of the standard ration. Additionally, shrimp offered the 10% bacterial biomass diet at 115% of the standard ration exhibited a significantly small FCR, implying that the bacterial biomass allows for better nutrient assimilation and conversion into growth.

### 4.3. Hemolymph Analysis

Hemolymph analysis data indicate a significant effect of bacterial biomass inclusion in diets on alkaline phosphatase activity. The hemolymph enzyme activity seems to increase with the inclusion of the bacterial biomass at 2.5%, only to decrease with further inclusion of the biomass. The significantly lowest enzyme activity corresponded to the highest inclusion level of the bacterial biomass. The bacterial biomass used in the present work is rich in minerals and contains around 4% phosphorus [20]. The increase in bacterial biomass inclusion contributes to the increase in the total phosphorus content in the diets. Alkaline phosphatase is an extracellular enzyme responsible for the breakdown of a variety of phosphorus‐containing compounds [43]. Alkaline phosphatase activity tends to increase with the increase in dietary phosphorus levels until sufficient levels are achieved. In the case of excess dietary phosphorus levels, alkaline phosphatase activity tends to decrease [44]. Similar results were obtained with sea cucumbers (*Apostichopus japonicus*), Chinese mitten crabs (*Eriocheir sinensis*), and grass carp (*Ctenopharyngodon idella*) offered diets with increasing levels of phosphorus [45–47].

### 4.4. Digestive Enzyme Gene Expression Analysis

Trypsin and chymotrypsin are the most abundant proteases in shrimp hepatopancreas [48]. Activity of both enzymes is influenced by a variety of factors, including but not limited to shrimp feeding behavior [49], feeding frequency [50], and protein level in the feed [51]. In the present work, the activity of both *trypsin* and *chymotrypsin* genes extracted from the hepatopancreas varied among dietary treatments. Despite not being statistically significant, the gene expression data obtained indicate that the inclusion of the bacterial biomass at 2.5% and 7.5% seems to positively influence the activity of proteases. However, a drop‐off in gene expression is observed when shrimp were offered the 10% bacterial biomass diet. Our results contradict the findings of Mendoza‐Porras et al. [18], who indicated that the inclusion of the same bacterial biomass at 10% of the diet of black tiger shrimp regulates digestive pathways. The authors observed the upregulation of several proteolytic enzymes, with trypsin and chymotrypsin being among them. Further analysis is needed to assess the reasons behind the decrease in both enzyme regulation in Pacific white shrimp offered 10% bacterial biomass in the diet.

### 4.5. Whole Body Proximate Analysis

The inclusion of bacterial biomass in shrimp diets significantly affected nutrient retention. Shrimp offered in the various diets retained more protein when the feed contained the bacterial biomass. The variation in protein retention cannot be attributed to a variation in the protein level of the feed, as all diets were formulated to be isonitrogenous and had similar protein levels when analyzed. Simon et al. [19] indicated that including the same bacterial biomass at 10% of black tiger shrimp diets allowed for better protein retention. Mendoza‐Porras et al. [18] explain that such dietary inclusion of the bacterial biomass positively impacts a variety of metabolic pathways. A variety of metabolic pathways involving amino acids were positively impacted, which in turn improved protein retention. We observed an increasing trend in ANPR, with the highest protein retention being observed in shrimp‐offered diets containing 10% bacterial biomass, regardless of the ration. Accordingly, our results are in accordance with Mendoza‐Porras et al. [18] and Simon et al. [19].

## 5. Conclusion

Our data support the use of the present bacterial biomass at 10% of Pacific white shrimp diets as the biomass improves shrimp performance in a variety of aspects. Considering that the product is proprietary, the exact mechanism of action remains unknown. However, the present data indicates that the bacterial biomass improves shrimp feed consumption and growth and, most importantly, results in low FCRs, indicating good feed utilization. Additionally, the bacterial biomass allows for an improvement in protein and phosphorus retention, which indicates an efficacious transformation of nutrients into growth and strong exoskeletons. Incorporating this bacterial biomass at 10% of shrimp diets helps with obtaining good shrimp growth with reduced FCRs.

## Funding

This research is supported by the Hatch Fund of the USDA’s National Institute of Food and Agriculture (Grant ALA016‐08027) and the Ridley Agriproducts Pty LTD.

## Conflicts of Interest

The authors declare no conflicts of interest.

## Data Availability

The data are made available upon reasonable request from the corresponding author.

## References

[1] Bai S. C. , Hamidoghli A. , and Bae J. , Feed Additives: An Overview, Feed and Feeding Practices in Aquaculture, 2022, Elsevier, 195–229, 10.1016/B978-0-12-821598-2.00015-1.

[2] Sathishkumar G. , Bhavatharaniya U. , Felix N. , Ranjan A. , and Prabhu E. , Strategies to Reduce Feed Cost by Improving Gut Health and Nutrient Utilisation of Fish in Aquaculture, Aquaculture. (2021) 25, no. 1.

[3] Zhang J. , Yang H. , Yan Y. , Zhang C. , Ye J. , and Sun Y. , Effects of Fish Origin Probiotics on Growth Performance, Immune Response and Intestinal Health of Shrimp (*Litopenaeus vannamei*) Fed Diets With Fish Meal Partially Replaced by Soybean Meal, Aquaculture Nutrition. (2020) 26, no. 4, 1255–1265, 10.1111/anu.13081.

[4] Sookying D. and Davis D. A. , Pond Production of Pacific White Shrimp (*Litopenaeus vannamei*) Fed High Levels of Soybean Meal in Various Combinations, Aquaculture. (2011) 319, no. 1-2, 141–149, 10.1016/j.aquaculture.2011.06.049.

[5] Chou R. L. , Her B. Y. , Su M. S. , Hwang G. , Wu Y. H. , and Chen H. Y. , Substituting Fish Meal With Soybean Meal in Diets of Juvenile Cobia *Rachycentron canadum* , Aquaculture. (2004) 229, no. 1–4, 325–333, 10.1016/S0044-8486(03)00395-8.

[6] Guo J. , Guo B. , Zhang H. , Xu W. , Zhang W. , and Mai K. , Effects of Nucleotides on Growth Performance, Immune Response, Disease Resistance and Intestinal Morphology in Shrimp *Litopenaeus vannamei* Fed With a Low Fish Meal Diet, Aquaculture International. (2016) 24, no. 4, 1007–1023, 10.1007/s10499-015-9967-7.

[7] Wang J. , Zhang H. , and Yang Q. , et al.Effects of Replacing Soybean Meal With Cottonseed Meal on Growth, Feed Utilization and Non-Specific Immune Enzyme Activities for Juvenile White Shrimp, *Litopenaeus vannamei* , Aquaculture Reports. (2020) 16, 10.1016/j.aqrep.2019.100255, 100255.

[8] Yue Y.-R. , Liu Y.-J. , and Tian L.-X. , et al.The Effect of Dietary Taurine Supplementation on Growth Performance, Feed Utilization and Taurine Contents in Tissues of Juvenile White Shrimp (*Litopenaeus vannamei*, Boone, 1931) Fed With Low-Fishmeal Diets, Aquaculture Research. (2013) 44, no. 8, 1317–1325, 10.1111/j.1365-2109.2012.03135.x.

[9] Galkanda-Arachchige H. and Davis D. A. , Evaluation of Differently Processed Soybean Meal Products as Ingredients in Practical Diets for Pacific White Shrimp *Litopenaeus vannamei* , Aquaculture Nutrition. (2020) 26, no. 2, 287–295, 10.1111/anu.12989.

[10] Dawood M. A. O. , Koshio S. , and Esteban M.Á. , Beneficial Roles of Feed Additives as Immunostimulants in Aquaculture: A Review, Reviews in Aquaculture. (2018) 10, no. 4, 950–974, 10.1111/raq.12209.

[11] Mente E. , Karalazos V. , Karapanagiotidis I. T. , and Pita C. , Nutrition in Organic Aquaculture: An Inquiry and a Discourse: Nutrition in Organic Aquaculture, Aquaculture Nutrition. (2011) 17, no. 4, e798–e817, 10.1111/j.1365-2095.2010.00846.x.

[12] De Jesus Raposo M. , De Morais A. , and De Morais R. , Marine Polysaccharides From Algae With Potential Biomedical Applications, Marine Drugs. (2015) 13, no. 5, 2967–3028, 10.3390/md13052967. 25988519 PMC4446615

[13] Ju Z. Y. , Forster I. , Conquest L. , and Dominy W. , Enhanced Growth Effects on Shrimp (*Litopenaeus vannamei*) From Inclusion of Whole Shrimp Floc or Floc Fractions to a Formulated Diet, Aquaculture Nutrition. (2008) 14, no. 6, 533–543, 10.1111/j.1365-2095.2007.00559.x.

[14] Van Doan H. , Prakash P. , and Hoseinifar S. H. , et al.Marine-Derived Products as Functional Feed Additives in Aquaculture: A Review, Aquaculture Reports. (2023) 31, 10.1016/j.aqrep.2023.101679, 101679.

[15] Vijayaram S. , Sun Y.-Z. , Zuorro A. , Ghafarifarsani H. , Van Doan H. , and Hoseinifar S. H. , Bioactive Immunostimulants as Health-Promoting Feed Additives in Aquaculture: A Review, Fish & Shellfish Immunology. (2022) 130, 294–308, 10.1016/j.fsi.2022.09.011. 36100067

[16] Glencross B. , Irvin S. , Arnold S. , Blyth D. , Bourne N. , and Preston N. , Effective use of Microbial Biomass Products to Facilitate the Complete Replacement of Fishery Resources in Diets for the Black Tiger Shrimp, *Penaeus monodon* , Aquaculture. (2014) 431, 12–19, 10.1016/j.aquaculture.2014.02.033.

[17] Glencross B. , Arnold S. , and Irvin S. , Bioactive Factors in Microbial Biomass Have the Capacity to Offset Reductions in the Level of Protein in the Diet of Black Tiger Shrimp, *Penaeus monodon* , Aquaculture. (2015) 446, 74–79, 10.1016/j.aquaculture.2015.04.007.

[18] Mendoza-Porras O. , Broadbent J. A. , and Beale D. J. , et al.Post-Prandial Response in Hepatopancreas and Haemolymph of *Penaeus monodon* Fed Different Diets. Omics Insights Into Glycoconjugate Metabolism, Energy Utilisation, Chitin Biosynthesis, Immune Function, and Autophagy, Comparative Biochemistry and Physiology Part D: Genomics and Proteomics. (2023) 46, 10.1016/j.cbd.2023.101073, 101073. 37018937

[19] Simon C. J. , Truong H. H. , Noble T. H. , Osborne S. A. , Wynne J. W. , and Wade N. M. , Microbial Biomass, Marine Invertebrate Meals and Feed Restriction Influence the Biological and Gut Microbiota Response of Shrimp *Penaeus monodon* , Aquaculture. (2020) 520, 10.1016/j.aquaculture.2019.734679, 734679.

[20] Simon C. , Briggs M. , and Truong H. , et al.The Science Behind NovaqPro, A Natural Functional Feed Additive for Prawns [Oral Presentation], *World Aquaculture 2023*, 2023, Darwin, Northern Territory, Australia, http://hdl.handle.net/102.100.100/487898?index=1.

[21] Truong H. H. , Hines B. M. , Rombenso A. N. , and Simon C. J. , Feed Intake, Gastro-Intestinal Transit and Haemolymph Free Amino Acids in the Shrimp *Penaeus monodon* Are Influenced by Marine Meal Supplementation, Aquaculture. (2021) 533, 10.1016/j.aquaculture.2020.736171, 736171.

[22] Peixoto S. , Soares R. , Silva J. F. , Hamilton S. , Morey A. , and Davis D. A. , Acoustic Activity of *Litopenaeus vannamei* Fed Pelleted and Extruded Diets, Aquaculture. (2020) 525, 10.1016/j.aquaculture.2020.735307, 735307.

[23] Peixoto S. , Strebel L. , Soares R. , and Davis D. A. , Acoustic Feeding Responses Using Marine Chemoattractants in Plant-Based Diets for Naive and Non-Naive *Litopenaeus vannamei* , Applied Animal Behaviour Science. (2022) 257, 10.1016/j.applanim.2022.105792, 105792.

[24] Soares R. , Peixoto S. , Davis R. P. , and Davis D. A. , Feeding Behavior and Growth of *Litopenaeus vannamei* Fed Soybean-Based Diets With Added Feeding Effectors, Aquaculture. (2021) 536, 10.1016/j.aquaculture.2021.736487, 736487.

[25] Tabbara M. , Strebel L. , Peixoto S. , Soares R. , Morais S. , and Davis D. A. , Use of Passive Acoustic Monitoring to Evaluate the Effects of a Feed Effector on Feeding Behavior, Growth Performance, and Salinity Stress Tolerance of *Litopenaeus vannamei* , Aquaculture. (2024) 582, 10.1016/j.aquaculture.2023.740499, 740499.

[26] Roy L. A. , Davis D. A. , Nguyen T. N. , and Saoud I. P. , Supplementation of Chelated Magnesium to Diets of the Pacific White Shrimp, *Litopenaeus vannamei*, Reared in Low-Salinity Waters of West Alabama, Journal of the World Aquaculture Society. (2009) 40, no. 2, 248–254, 10.1111/j.1749-7345.2009.00247.x.

[27] Liu W. G. , Zhang J. R. , Cao Z. Q. , Xu F. Y. , and Yao K. D. , A Chitosan-Arginine Conjugate as a Novel Anticoagulation Biomaterial, Journal of Materials Science: Materials in Medicine. (2004) 15, no. 11, 1199–1203, 10.1007/s10856-004-5672-1. 15880928

[28] Xie S. , Wei D. , and Fang W. , et al.Survival and Protein Synthesis of Post-Larval White Shrimp, *Litopenaeus vannamei* Were Affected by Dietary Protein Level, Animal Feed Science and Technology. (2020) 263, 10.1016/j.anifeedsci.2020.114462, 114462.

[29] Ulaje S. A. , Rojo-Arreola L. , Lluch-Cota S. E. , Ascencio F. , Cruz-Hernández P. , and Sicard M. T. , Gene Expression and Energetic Metabolism Changes in the Whiteleg Shrimp (*Litopenaeus vannamei*) in Response to Short-Term Hypoxia, Aquaculture Research. (2019) 50, 10.1111/are.13960, are.13960.

[30] Livak K. J. and Schmittgen T. D. , Analysis of Relative Gene Expression Data Using Real-Time Quantitative PCR and the 2−ΔΔCT Method, Methods. (2001) 25, no. 4, 402–408, 10.1006/meth.2001.1262. 11846609

[31] Shapiro S. S. and Wilk M. B. , An Analysis of Variance Test for Normality (Complete Samples), Biometrika. (1965) 52, no. 3-4, 591–611, 10.1093/biomet/52.3-4.591.

[32] White H. , A Heteroskedasticity-Consistent Covariance Matrix Estimator and a Direct Test for Heteroskedasticity, Econometrica. (1980) 48, no. 4, 10.2307/1912934, 817.

[33] Bartlett M. S. , Properties of Sufficiency and Statistical Tests, Proceedings of the Royal Society of London. Series A - Mathematical and Physical Sciences. (1937) 160, no. 901, 268–282, 10.1098/rspa.1937.0109.

[34] Derveaux S. , Vandesompele J. , and Hellemans J. , How to Do Successful Gene Expression Analysis Using Real-Time PCR, Methods. (2010) 50, no. 4, 227–230, 10.1016/j.ymeth.2009.11.001. 19969088

[35] Smith D. V. and Shahriar M. S. , A Context Aware Sound Classifier Applied to Prawn Feed Monitoring and Energy Disaggregation, Knowledge-Based Systems. (2013) 52, 21–31, 10.1016/j.knosys.2013.05.007.

[36] Smith D. V. and Tabrett S. , The use of Passive Acoustics to Measure Feed Consumption by *Penaeus monodon* (Giant Tiger Prawn) in Cultured Systems, Aquacultural Engineering. (2013) 57, 38–47, 10.1016/j.aquaeng.2013.06.003.

[37] Darodes de Tailly J. , Keitel J. , Owen M. A. G. , Alcaraz-Calero J. M. , Alexander M. E. , and Sloman K. A. , Monitoring Methods of Feeding Behaviour to Answer Key Questions in Penaeid Shrimp Feeding, Reviews in Aquaculture. (2021) 13, no. 4, 1828–1843, 10.1111/raq.12546.

[38] Eap D. , Correa S. , Ngo-Vu H. , and Derby C. D. , Chemosensory Basis of Feeding Behavior in Pacific White Shrimp, *Litopenaeus vannamei* , The Biological Bulletin. (2020) 239, no. 2, 115–131, 10.1086/710337. 33151752

[39] Tantikitti C. , Feed Palatability and the Alternative Protein Sources in Shrimp Feed, Songklanakarin Journal of Science and Technology. (2014) 36, no. 1, 51–55.

[40] Sanchez D. R. , Fox J. M. , Lawrence A. L. , Castille F. L. , and Dunsford B. , A Methodology for Evaluation of Dietary Feeding Stimulants for the Pacific White Shrimp, *Litopenaeus vannamei* , Journal of the World Aquaculture Society. (2007) 36, no. 1, 14–23, 10.1111/j.1749-7345.2005.tb00126.x.

[41] NRC (with Studies, Division on Earth and Life, Resources, Board on Agriculture and Natural, & Shrimp, Committee on the Nutrient Requirements of Fish and) , Nutrient Requirements of Fish and Shrimp, 2011, 1st edition, National Academies Press.

[42] Rombenso A. N. , Duong M. H. , Hines B. M. , Mã T. , and Simon C. J. , The Marine Microbial Biomass, Novacq, a Useful Feed Additive for Postlarvae and Juvenile *Litopenaeus vannamei* , Aquaculture. (2021) 530, 10.1016/j.aquaculture.2020.735959, 735959.

[43] Wang Y. and He Z. , Effect of Probiotics on Alkaline Phosphatase Activity and Nutrient Level in Sediment of Shrimp, *Penaeus vannamei*, Ponds, Aquaculture. (2009) 287, no. 1-2, 94–97, 10.1016/j.aquaculture.2008.10.022.

[44] Wang Y. , Geng Y. , and Shi X. , et al.Effects of Dietary Phosphorus Levels on Growth Performance, Phosphorus Utilization and Intestinal Calcium and Phosphorus Transport-Related Genes Expression of Juvenile Chinese Soft-Shelled Turtle (*Pelodiscus sinensis*), Animals. (2022) 12, no. 22, 10.3390/ani12223101, 3101. 36428331 PMC9687074

[45] Lei Y. , Sun Y. , and Wang X. , et al.Effect of Dietary Phosphorus on Growth Performance, Body Composition, Antioxidant Activities and Lipid Metabolism of Juvenile Chinese Mitten Crab (*Eriocheir sinensis*), Aquaculture. (2021) 531, 10.1016/j.aquaculture.2020.735856, 735856.

[46] Song Z. , Li P. , Hu S. , Liu C. , Hao T. , and Han X. , Influence of Dietary Phosphorus on the Growth, Feed Utilization, Proximate Composition, Intestinal Enzymes, and Oxidation Resistance of Sea Cucumber *Apostichopus japonicus* , Aquaculture Nutrition. (2023) 2023, 1–12, 10.1155/2023/2266191, 2266191. PMC10139806 37124880

[47] Wen J. , Jiang W. , and Feng L. , et al.The Influence of Graded Levels of Available Phosphorus on Growth Performance, Muscle Antioxidant and Flesh Quality of Young Grass Carp (*Ctenopharyngodon idella*), Animal Nutrition. (2015) 1, no. 2, 77–84, 10.1016/j.aninu.2015.05.004. 29767010 PMC5884464

[48] Dall W. , Feeding, Digestion and Assimilation in Penaeidae, *Proceedings of the Aquaculture Nutrition Workshop*, 1992, 57–63.

[49] Navarrete Del Toro M. A. and García-Carreño F. , The Toolbox for Protein Digestion in Decapod Crustaceans: A Review, Reviews in Aquaculture. (2019) 11, no. 4, 1005–1021, 10.1111/raq.12276.

[50] Hernandez-Cortes P. , Quadros-Seiffert W. , del Toro M. A. N. , Portillo G. , Colado G. , and Garcia-Carreño F. L. , Rate of Ingestion and Proteolytic Activity in Digestive System of Juvenile White Shrimp, *Penaeus vannamei*, During Continual Feeding, Journal of Applied Aquaculture. (1999) 9, no. 1, 35–45, 10.1300/J028v09n01_03.

[51] Sainz J. C. , García-Carreño F. L. , Córdova-Murueta J. H. , and Cruz-Hernández P. , Whiteleg Shrimp (*Litopenaeus vannamei*, Boone, 1931) Isotrypsins: Their Genotype and Modulation, Journal of Experimental Marine Biology and Ecology. (2005) 326, no. 1, 105–113, 10.1016/j.jembe.2005.05.021.

